# An overview of the perspectives used in health economic evaluations

**DOI:** 10.1186/s12962-024-00552-1

**Published:** 2024-05-14

**Authors:** Manit Sittimart, Waranya Rattanavipapong, Andrew J. Mirelman, Trinh Manh Hung, Saudamini Dabak, Laura E. Downey, Mark Jit, Yot Teerawattananon, Hugo C. Turner

**Affiliations:** 1 Health Intervention and Technology Assessment Program (HITAP), Nonthaburi, Thailand; 2 World Health Organization, Geneva, Switzerland; 3 Oxford University Clinical Research Unit (OUCRU), Ho Chi Minh City, Vietnam; 4 The George Institute for Global Health, School of Public Health, Imperial College London, London, UK; 5 The George Institute for Global Health, University of New South Wales, Sydney, Australia; 6 Department of Infectious Disease Epidemiology, London School of Hygiene and Tropical Medicine, London, UK; 7 School of Public Health, University of Hong Kong, Hong Kong Special Administrative Region, Hong Kong, China; 8 Saw Swee Hock School of Public Health, National University of Singapore (NUS), Singapore, Singapore; 9 MRC Centre for Global Infectious Disease Analysis, School of Public Health, Imperial College London, London, UK

**Keywords:** Perspective, Definition, Economic evaluation, Health economics, Global health, LMIC, Societal

## Abstract

The term ‘perspective’ in the context of economic evaluations and costing studies in healthcare refers to the viewpoint that an analyst has adopted to define the types of costs and outcomes to consider in their studies. However, there are currently notable variations in terms of methodological recommendations, definitions, and applications of different perspectives, depending on the objective or intended user of the study. This can make it a complex area for stakeholders when interpreting these studies. Consequently, there is a need for a comprehensive overview regarding the different types of perspectives employed in such analyses, along with the corresponding implications of their use. This is particularly important, in the context of low-and-middle-income countries (LMICs), where practical guidelines may be less well-established and infrastructure for conducting economic evaluations may be more limited. This article addresses this gap by summarising the main types of perspectives commonly found in the literature to a broad audience (namely the patient, payer, health care providers, healthcare sector, health system, and societal perspectives), providing their most established definitions and outlining the corresponding implications of their uses in health economic studies, with examples particularly from LMIC settings. We then discuss important considerations when selecting the perspective and present key arguments to consider when deciding whether the societal perspective should be used. We conclude that there is no one-size-fits-all answer to what perspective should be used and the perspective chosen will be influenced by the context, policymakers'/stakeholders’ viewpoints, resource/data availability, and intended use of the analysis. Moving forward, considering the ongoing issues regarding the variation in terminology and practice in this area, we urge that more standardised definitions of the different perspectives and the boundaries between them are further developed to support future studies and guidelines, as well as to improve the interpretation and comparison of health economic evidence.

## What is the perspective in health economic evaluations?

Health economic analyses, particularly economic evaluations and costing studies, have an important role in investigating the value-for-money of health interventions and supporting decision-making surrounding resource allocation within the health sector [1–3]. Such studies are a key element of Health Technology Assessment (HTA) processes and other priority-setting or decision-making processes [1, 4, 5]. When conducting an economic evaluation of a particular health intervention or technology, understanding the perspective, or the point of view from which the evaluation is conducted is important, as it determines the boundary of the study and which types of costs and consequences/outcomes are included within the analysis [6]. Note that cost is a general term that refers to the value of resources/inputs used to produce a good or service. As different perspectives include (or exclude) different costs and outcomes, they can substantially influence the results of health economic studies and the subsequent recommendations and policies informed by these studies [7]. Therefore, it is vital that the perspective is carefully considered when conducting, reviewing, or interpreting health economic analyses.

Different types of perspectives have been adopted in health economic studies. However, there is no universally accepted “right” answer regarding which perspective should be applied, and this decision will depend on the context, type of analysis, decision-maker and question that the evaluation aims to answer [7]. Due to contextual considerations, the perspective is one of the methodological areas that exhibits the largest variation within the currently available guidelines for health economic studies [8–10]. Therefore, this is a potentially challenging area for stakeholders when conducting and/or interpreting these studies. Consequently, there is a need for an overview outlining the key types of perspectives, along with the corresponding implications of using different perspectives in health economic analyses. This is particularly important in the context of low-and-middle-income countries (LMICs), where there are less well-established guidelines and infrastructure (including data) for conducting economic evaluations and subsequently the potential for more variation in methodology between studies. To date, the Guide to Economic Analysis and Research (GEAR) resource has only identified 14 national guidelines from LMICs related to conducting health economic evaluations [11].

This article aims to outline and introduce the main types of perspectives used in economic evaluations, as well as to discuss their implications on cost-effectiveness calculations. We also outline ongoing issues and considerations related to perspectives that are important to be aware of when comparing and interpreting economic evaluations. It is expected that greater awareness of these concepts will lead to better consistency in future health economic studies and improve the interpretation and comparison of health economic evidence.

## What are the main types of perspectives?

Here we provide a breakdown of the most commonly used perspectives within health economic evaluations and costing studies. These are derived from a review of key texts [8, 12, 13], and recommendations from multiple international and national economic evaluation guidelines listed in the International Society for Pharmacoeconomics and Outcomes Research (ISPOR) and Guide to Economic Analysis and Research (GEAR) websites [11, 14, 15]. That said, it is important to note that there is variation in terminology used within the field to describe perspectives as well as other terms for perspectives not included here. We have endeavoured to highlight what we consider to be the most established definitions.

**Fig. 1:**
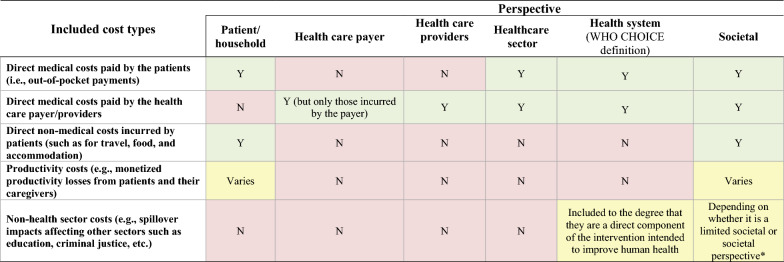
Overview of the different perspectives and the variation of included costs within economic evaluations. Y: Included; N: Not included; * The limited societal perspective excludes spillover impacts affecting sectors other than health care whereas the (non-limited) societal perspective includes the spillover impacts on at least one non-health care sector. Note that there is variation in terminology used within the field to describe these different perspectives as well as others not included here. Therefore, it is possible some studies would apply these perspectives differently to what we have outlined

The differences between these perspectives relate to what cost (and cost saving) items may be included within an analysis. Figure 1 provides an overview of the different perspectives and the variation of included costs. In the context of cost-effectiveness and cost-utility analysis, the inclusion of non-monetary health outcomes (such as disability-adjusted life year (DALY), quality-adjusted life year (QALY) or cases averted) in the denominator of the cost-effectiveness ratio calculations would not typically be influenced by these perspectives whereas the costs in the numerator would be directly influenced.

### Patient/household

The patient or household perspectives are used to describe the costs borne by individuals or their households, respectively. The patient perspective may be limited to the costs incurred by the patient whereas the household perspective also includes the costs incurred by other members of the household. This distinction is not always made as the terms are at times used interchangeably. Under the patient/household perspective, all costs that patients incur when facing a health issue could be included, such as direct medical costs that are not covered by the patient's health insurance (i.e., out-of-pocket costs, co-payments, and deductibles), direct non-medical costs (such as those from transport to health facilities), and potentially productivity costs—also known as indirect costs (i.e., monetised productivity losses resulting from lost paid and unpaid work due to an illness or an intervention). The extent to which these cost types are included depends on the type of study being conducted. Within the context of an economic evaluation of a health system intervention, all costs borne by the patient would be included. While the patient/household perspective is more likely to be used within cost-of-illness studies or analysis of patient health expenditure/analyses of financial risk protection [16], it is rarely applied within full economic evaluations. However, some have advocated for using this perspective in economic evaluations in the context of the increasing focus on patient-centred outcomes in health policy research [8, 12].

### Health care payer or payer

The health care payer perspective includes costs incurred by specific health care payer(s)—typically a third party, such as a specific health organisation, specific control programme or agency that manages an insurance programme [9]. This would include the costs incurred by a specific health care payer related to treatment, disease management or other health care services [9, 16]. However, the costs that are not borne by this specific payer will not be considered (such as the out-of-pocket payments paid by patients). It is important to note that the payer perspective would only relate to the part of the organisation that the funds have been planned or budgeted for [17]. Therefore, it would capture the costs incurred by a specific control programme but not the costs incurred by the broader health care provider(s). It is noteworthy that in some settings there can be multiple relevant payers (such as multiple insurance programmes).

### Health care provider(s)

The health care provider’s perspective will include all costs incurred by a given provider (or group of providers in the health system) in delivering care services to patients. Depending on the context, this can be the same as the health care payer perspective. However, the health care provider(s) perspective is usually broader in terms of its scope of costs included; as the payer perspective only relates to the specific part of the organisation that the funds have been planned/budgeted for [17]. The difference between the payer and provider perspectives will depend on the context of the study, but it is plausible that the provider’s perspective will give a more complete picture of total costs and hence is used more often in costing exercise [16].

### Healthcare sector

The healthcare sector perspective is similar to the health care provider(s) perspective but broader and accounts for all the costs directly associated with the healthcare sector, regardless of who will bear such costs. This means that it not only includes the direct medical costs incurred by specific third-party payers (such as national health services), but it also includes the out-of-pocket payments for health care made by patients [9]. Costs that are not directly related to medical services/the health sector are considered outside of the scope of the healthcare sector perspective—such as costs related to the patients' travel or accommodation and productivity costs (indirect costs) [18]. The distinction between health care payer/provider and healthcare sector perspectives may be particularly important in LMIC settings where out-of-pocket payments by patients can be a significant source of health care expenditure [19].

### Health system

The definition of the health system perspective is more variable within the literature [20–24]. The latest WHO-CHOICE guidelines [20, 21] defined the health system perspective as including an ensemble of actions and actors whose primary intent is to improve human health. This therefore includes all direct, market-valued costs, whether public or private, that are required to deliver the intervention, regardless of payer. This would also cover the out-of-pocket payments for health care made by patients but would not account for the patients’ direct non-medical costs (such as travel-related costs), and productivity costs. This definition is subtly broader than the healthcare sector perspective (Fig. 1), as it can potentially include costs from other sectors when they are a direct component of the intervention intended to improve human health (e.g., the costs associated with developing health legislation and costs associated with regulation of health care and products) [20]. It is debatable how often these are included. Costs outside of the health system that are not primarily health oriented would not be included. It should be noted that in some cases, it is possible that the term health system perspective is being defined differently—such as to refer to the costs incurred by a particular publicly funded national healthcare provider. If this was the case, it could be more equivalent to the use of the health care provider(s) perspective as outlined above.

### Societal

The societal perspective is the broadest and includes all healthcare-related costs, regardless of who is paying, including the patients'/caregivers’ costs for accessing an intervention (such as for travel and accommodation etc.) and their productivity costs [9]. This perspective can also potentially include other “relevant” non-health-related impacts in other sectors [25] such as those on social services, education, legal or criminal justice, environment, etc. In practice, there is variation in how far the societal perspective is taken and whether the impact on other sectors is included [13]. Kim et al. stratified the societal perspective by whether it is limited or not (Fig. 1) [9]. The limited societal perspective includes all healthcare-related costs (including the patients' costs) but excludes spillover impacts affecting sectors other than health care. In contrast, the (non-limited) societal perspective is broader and also includes the cost impacts on at least one non-healthcare sector [9]. In macroeconomic models, the societal perspective would also include the sectoral impact on other sectors due to changes in demand and supply in the economy [26, 27].

It should be noted that there will be other types of perspectives not captured here that can fall in between these categories. For example, the National Institute for Health and Care Excellence (NICE) in the UK recommends “The perspective adopted on costs should be that of the NHS and personal social services.” [28]. This would be broader than the health system perspective as defined here as it includes the social care related costs, but not as broad as the societal perspective.

Some guidelines now recommend the use of a disaggregated societal perspective [29, 30], where the costs and outcomes are disaggregated, either by sector of the economy or by who incurs them—and therefore it is possible to interest the results from a range of perspectives.

It should be noted that there are types of costs that may be excluded from the societal perspective [22]. For example, some interventions may result in transfer costs or payments; financial flows from one part of society to another, that do not consume resources but simply transfer the power to use resources from one person or sector to another (such as import tariffs as well as unemployment or sickness benefits) [31]. Transfer payments can be a cost to the paying government or control programme, but a financial gain to another sector or a patient. Therefore, because they do not use or create resources, transfer payments are typically not considered when estimating economic costs using a societal perspective [22, 32], but can be included when using a narrower perspective (such as the health care payer perspective).

## Implications on economic evaluations

There are several implications of the study perspective on health economic evaluations. The first is the scope of costs related to the intervention that are included. Generally, in terms of the cost of the intervention, the broader the perspective the higher the potential cost of the intervention (the impact will depend on the context). For example, when looking at the costs of providing a vaccine at a health clinic, the health care provider(s) perspective would only include the costs that are incurred by the government’s health service (such as those associated with the staff’s time, and the purchase of the vaccine etc.). However, under the societal perspective, the costs that are incurred by the patients in order to go to the clinic and get the vaccine would also be included (such as their travel costs and potentially their productivity costs associated with lost paid or unpaid work), increasing the overall cost of the intervention. Similarly, for cost-of-illness studies, the broader the perspective the wider the scope of costs included.

A related implication is that the choice of study perspective can have a significant impact on both the source of cost data and the method used for data collection. For example, this determines whether patient interviews may be needed. Having different sources of cost data and the way they are collected can contribute to the variation of cost values included in studies. For example, the costs for treating a patient at a hospital may be based on 'reimbursement rates’ under the payer perspective whereas the full cost of the resources utilised may be used under the health care provider perspective.

It is also important to note that, theoretically, the chosen perspective of an economic evaluation should not influence whether financial or economic costs should be adopted [33]. Nonetheless, the chosen perspective can influence how economic costs are valued and whether adjustments to market prices are required [33]. For example, if adopting the health care provider or payer perspective, it might be appropriate to use the market prices of a drug or vaccine that the provider has procured. However, if using the societal perspective, these prices may need to be adjusted to reflect their social opportunity costs (their value in their next best alternative use—only reflecting their short-run manufacturing and distribution costs), rather than their market price [22, 34].

A further implication within economic evaluations is that the perspective will determine the consequences/outcomes that are included. A key example is that it affects if/what “cost savings” or cost offsets are included within the analysis. These “cost savings” are effectively deducted from the intervention cost within the cost-effectiveness ratios. These cost savings could include the costs associated with disease cases that are averted due to the intervention (for example in the case of measles vaccination, they would consider the cost savings associated with the averted measles cases that it prevents—such as the medical costs associated with hospitalised cases). The broader the perspective, the broader the types of costs included within these savings, and with the societal perspective, it can include prevented productivity costs that would have been associated with the morbidity and mortality of the cases and even costs outside the healthcare sector.

A further implication of the perspective is regarding the inclusion of future unrelated costs within these analyses [35–39]. Health interventions can increase the life expectancy of patients and consequently influence the consumption of both unrelated medical and non-medical resources during the additional lifetime they generate. These future unrelated costs are typically grouped into future medical costs (e.g., the costs of treating people with other future unrelated health conditions) and future non-medical costs (the costs related to consumption of non-medical resources, such as food, housing, utilities etc.). Which types of future unrelated costs that could be included within an economic evaluation, would be influenced by the perspective (with the societal perspective potentially including both future unrelated medical and non-medical costs). If these future costs are taken into account, adopting a broader perspective, could lead to a greater increase in the net cost of the intervention when it impacts the patient’s survival. This can therefore subsequently impact the estimated cost-effectiveness of the intervention. There is currently ongoing debate about the inclusion of these future unrelated costs in health economic analyses [35–39]. This debate and variation should be considered when interpreting different studies. The inclusion of future unrelated costs is still uncommon, and further guidance on this area is needed [35].

A particular area of debate is regarding the inclusion of future unrelated medical costs [35]. A key issue here is that the costs and outcomes of unrelated events in the future will depend on decisions not yet made and are therefore difficult to predict. This debate and variation should be considered when interpreting different studies.

Ultimately, although in some cases the use of different perspectives may only have a small impact on the cost-effectiveness ratios, it can also have a significant impact and could fundamentally change the conclusions of studies (Table 1). In some cases, broadening the perspective will not greatly change the estimated cost of the intervention, but could result in more cost-savings being included, resulting in the estimated cost-effectiveness ratio decreasing (Table 1). On the other hand, it is also possible that broadening the perspective would increase the cost of an intervention—potentially making its cost-effectiveness ratio increase (if this increase in the intervention cost outweighs any potential increase in the cost-savings). This relative impact and direction of the change on the cost-effectiveness ratio will depend on the context of the study and the intervention being investigated (Table 1). It should be noted that in some cases, health interventions may be estimated to be cost saving (i.e., have negative ICER values) even when using a more restricted perspective. For example, Owen et al. [40] found that among the cost-effectiveness analysis of public health interventions examined between 2005 to 2018 by the National Institute for Health and Care Excellence (NICE) in the UK, 21% were projected to generate cost savings even without using a societal perspective. In these cases, changing to a societal perspective would be unlikely to influence the results/policy recommendation. However, this will not always be the case and the perspective can have a significant impact (Table 1). This is particularly important to consider in countries in which the patients incur higher costs for assessing/receiving health care.

**Table 1:** Case studies regarding the impact of different perspectives on incremental cost-effectiveness ratios relating to interventions in Vietnam

Intervention and chosen perspective	Cost per DALY averted (US$)
Wolbachia deployments to control dengue (compared to the status quo) [41]	
Health care provider perspective	708.21
Health sector perspective	419.56
Societal perspective (excluding the productivity gains related to prevented excess mortality)	Dominates the comparator
Societal perspective	Dominates the comparator
Rotavirus vaccination (compared to no vaccination) [42]	
Healthcare system perspective	1777
Societal perspective (only including the productivity costs related to caregivers' time spent caring for sick children)	1744
*Haemophilus influenzae* type b (Hib) vaccination (compared to no vaccination) [43]	
Governmental perspective	1850
Societal perspective (excluding the productivity gains related to prevented excess mortality)	1662
*Values were adjusted for inflation to 2020 US$ (using GDP deflators pertaining to Vietnam *[44, 45]*)*	

Due to this variation, if studies have used different perspectives, a direct comparison of results may be misleading. Of concern, even when the same perspective is reported to be used, the variation in the specific cost items included (Table 1) could still negatively impact the comparability of studies. A key driver in the variation of cost-effectiveness ratios between the use of the societal and other perspectives, is the specific types of costs being considered and if/what types of productivity costs are being included. Notably, there are issues surrounding the inclusion of productivity costs and potential double counting (outlined in Box 1). This highlights the importance of considering the perspective when comparing studies and the need to clearly report methodology regarding productivity costs.

Box 1: Issues surrounding productivity costs within economic evaluations (adapted from [46])

**Table:** 

Within an economic evaluation, one of the main cost savings that could be included when using the societal perspective is related to productivity costs. However, there is variation in the type of productivity costs included and potential double-counting of outcomes within a cost-utility analysis. When using a societal perspective, valuing the productivity losses related to patients' time lost for accessing an intervention is relatively uncontroversial. However, it has been argued that valuing the productivity gains associated with the morbidity/mortality that the intervention prevents should not be included [34, 47–52]. This is because these benefits are arguably already being captured within the quality-adjusted life year (QALY) or disability-adjusted life year (DALY) outcome measure and therefore, including these monetised productivity gains as a cost saving would potentially be double-counting the outcomes of the intervention [53]. However, this recommendation has been challenged with some arguing that the QALY measure does not capture these productivity gains [34, 47–51]. Guidelines regarding the inclusion of these productivity gains vary [8, 54, 55]. Due to this, there is variation regarding the types of productivity costs included within economic evaluations, even when a societal perspective is used, and studies may show their results both with and without productivity costs or only including certain types of productivity costs It should also be noted that estimates of productivity costs are highly sensitive to the methodology used and are associated with a number of challenges [56]. Not only can quantifying productivity losses accurately be challenging, but the underlying approaches used for the quantification can also give very different results (such as the human capital approach vs the friction cost approach) [46]. A further source of variation to note is how these productivity losses are monetised [56]. The variation in how the productivity costs are quantified and valued leads to inconsistencies between studies [57], and it is important to be aware of this variation in methodology when comparing studies [46]

## What is used in practice?

A review of the perspectives used in costing in cost-effectiveness analysis between 1974–2018 has been conducted by Kim et al. [9]. Interestingly, they found that studies often misspecified or did not clearly state the perspective used. After re-classification by registry reviewers, they found that a healthcare sector or payer perspective was the most common (74%) and that cost-effectiveness analysis rarely included impacts on non-healthcare sectors [9].

In terms of the available national economic evaluation guidelines (including from high income countries), a cross-country comparison by Sharma et al. [13] found that of the 31 guidelines they reviewed, 15 (48%) recommended using one of the non-societal perspectives (such as payer, health care provider health sector, health system etc.). However, the corresponding terminology used to describe these perspectives was variable. Three guidelines (10%) stated that any perspective relevant to the research question may be considered. Eight guidelines (26%) recommended using the societal perspective for the primary analysis, and 10 (26%) recommended using the societal perspective for additional analysis if required [13]. Yet, Sharma et al. also highlighted that even when the societal perspective was recommended, there was variation regarding the specific recommendations on the type of costs that should be included [13]. For example, the guidelines for Portugal recommended that intangible costs should also be included under the societal perspective [58], whereas the guidelines for Norway recommended using a societal perspective but the inclusion of productivity costs was optional [59]. In addition, while several guidelines recommended including all costs and outcomes within and outside the healthcare sector, others recommended for the more limited societal perspective excluding the impacts of the intervention on non-healthcare sectors [13]. This highlights the notable variation surrounding the societal perspective. A recent review of how the societal perspective is defined within guidelines by Avşar et al. also found substantial variation of the definition, including insufficient guidance on what to include under different perspectives [8]. Among 46 guidelines included in their review, the societal perspective featured in 30 guidelines, of which 21 (70%) explicitly considered this perspective (at times it was recommended within additional analysis). In several guidelines where productivity costs were allowed in additional analysis, this was usually referred to as a broader perspective (than healthcare), instead of explicitly defining it as a societal perspective. Interestingly, countries with multiple payers in the health systems were more likely to consider the societal perspective.

Table 2 highlights the recommendations regarding what perspective to use within key international/LMIC economic evaluation guidelines. The national economic evaluation guidelines were extracted from GEAR [11] (please note that some guidelines were not included as their text was not available in English). The focus on LMICs in Table 2 was chosen because literature providing contextual insights/case studies from LMICs are typically limited (despite the need for increased capacity in these settings). In terms of international guidelines, the WHO-CHOICE 2003 guidelines on cost-effectiveness analysis recommended using the societal perspective but excluding productivity costs [55]. The WHO-CHOICE's latest guidelines have now adopted a health system perspective [21]. In contrast, the International Decision Support Initiative (iDSI) reference case for economic evaluation recommended using a disaggregated societal perspective (where the costs and outcomes are disaggregated, either by sector of the economy or by who incurs them, making it possible to interpret the results from a range of perspectives) [29, 30]. In terms of the available LMIC national economic evaluation guidelines, recommendations for the use of one of the non-societal perspectives were the most common. This could be because adopting these non-societal perspectives is relatively less complex and requires fewer data. That said, the societal perspective was recommended in several cases. In contrast, the perspective recommended for budget impact analysis is generally more consistent within guidelines, with the public payer or service purchaser perspectives typically recommended [60].

**Table 2:** The recommendations within several international and LMIC national economic evaluation guidelines on perspective to use within an economic evaluation

Sources	Recommendation
Key international guidelines	
WHO-CHOICE (2003) [55] WHO-CHOICE (2017 update) [20]	Societal perspective (but excluding productivity costs) Health system perspective
iDSI and Gates reference cases [29, 30]	Disaggregated societal perspective^a^
Country guidelines/reference cases^b^	
Thailand: Guideline for Health Technology Assessment in Thailand Updated Edition 2019 [61]	Societal perspective (health care provider perspective can be used as a secondary perspective)
India: Health Technology Assessment in India A Manual [62]	Disaggregated societal perspective
Egypt: Guidelines for Reporting Pharmacoeconomic Evaluations 2013 [63]	Healthcare system perspective
The Philippines: Philippine HTA Methods Guide—Methodological standards in evaluation of health technologies in the Philippines 1st edition 2020 [64]	Publicly funded health care payer perspective
Indonesia: Indonesian Health Technology Assessment Committee (InaHTAC) Guideline [65]	Both societal and provider perspectives are considered
Brazil: Methodological Guidelines: Economic Evaluation of Health Technologies 2014 [15]	Healthcare system perspective (service payer/purchaser). Societal perspective is also recommended as an additional analysis
Malaysia: Pharmacoeconomic guidelines 2nd edition 2019 [66]	Payer/budget holder perspective (in Malaysia’s context, this is the same as health care provider)
Republic of Ghana: Reference Case for Health Technology Assessment (HTA) 1st edition 2023 [11]	Societal perspective. However, the perspective could be that of the government (defined to include the public-funded health system or the National Health Insurance Authority)
Russia: Guidelines for conducting a comparative clinical and economic evaluation of drugs 2018 [14]	Health care payer perspectives
South African: Health Technology Assessment Methods Guideline v1.2. Essential Drugs Program (EDP) 2022 [11]	Public health system
Colombia: Manual para la elaboración de evaluaciones económicas en salud 2014 [67]	National health system perspective
China: China Guidelines for Pharmacoeconomic Evaluation (CGPE) 2020 [68]	Health system and societal as the primary perspectives
Mexico: Economic Assessment Study Guideline for Updating The National Formulary in Mexico 2017 [69]	Public health sector/institutions
Cuba: Methodological Guidelines for Health Economic Evaluation 2003 [70]	Societal, government, or patient perspectives

^a^Where the costs and outcomes are disaggregated, either by sector of the economy or by who incurs them—and therefore it is possible to interest the results from a range of perspectives

^b^The national economic evaluation guidelines were extracted from GEAR [11] (please note that some guidelines were not included as their text was not available in English)

## Selecting the perspective

In practice, it is important to note that there is no one-size-fits-all recommendation regarding what perspective should be used. The right perspective will depend on the research question, context, and goals of the decision-makers [7, 34, 71]. For example, if the goal is to understand the affordability of an intervention, the payer perspective may be the most appropriate.

When choosing the perspective, it is important to consider the role of patient out-of-pocket payments. Crucially, the payer and health care providers perspectives will not account for any costs paid by patients (including their out-of-pocket payments). They therefore may not be suitable for interventions that require co-payment by patients—as they will underestimate the cost of the intervention and potentially lead to inefficient policy recommendations. This is particularly important in a global heath context as patient out-of-pocket payments are one of the most critical healthcare funding sources in many LMICs [72]. In this context, at least the use of a healthcare sector perspective (if not a broader perspective) would be needed to account for these out-of-pocket payments (as outlined in Fig. 1).

### Key considerations regarding navigating the use of societal perspective

In terms of selecting perspective, it should be noted that there is ongoing debate regarding the role of the societal perspective and when it should be used. The societal perspective is often referred to as the gold standard for economic evaluations [73–75] and recommended in several guidelines. The reasons for this relate to the fact that it considers a more complete picture of costs and consequences/outcomes. This has important advantages in the context of evaluating health interventions and promoting total welfare and the good of society. For example, since the societal perspective considers a full set of information regarding conceivable costs and outcomes, it has been argued that it offers a higher level of decision-supportive power and will be less dependent on the study commissioners, as well as the political and social character of the society that the study is intended for [73, 74, 76–78]. A focus solely on the health care payer/provider perspective could overlook interventions that demonstrate cost-effectiveness from a broader societal standpoint. Furthermore, excluding important costs and outcomes within an economic evaluation, as seen in more restricted perspectives, could lead to inefficient resource allocation decisions [78]. The societal perspective can identify cost-shifting between sectors and on to patients/their families [78] (e.g., if the costs to the health systems are decreased but the costs to patients are increased), which may not be accounted for with more restricted perspectives. Consequently, many have argued that the societal perspective is preferable to others [73, 75, 78].

However, there are important further considerations that need to be made when considering the societal perspective—particularly in a global health context [7, 71]. Firstly, having an all-inclusive analysis from a societal perspective, where in theory all conceivable costs and outcomes are considered, may require more costs and effort in order to acquire the additional data and information. As such, there needs to be a balance between the costs of acquiring additional information needed to use the societal perspective and increasing the quality of the decision being made. In the context of having inadequate or inaccessible datasets (such as those related to epidemiology, resource uses, unit costs, baseline distribution of health outcomes and data to inform the cost-effectiveness threshold), there is a greater challenge to the adoption of a broader perspective [79]. This is the rationale why the proponents of adaptive HTA suggest that a more limited perspective can be used in more nascent systems [80]. Applying a narrower perspective, especially in the cases of limited data, may be more pragmatic, albeit presenting some degree of omitted variable bias.

Although the societal perspective has often been advocated for, less consideration has been given to what this should include and its practical implementation [74, 81]. In practice, it is not always easy to define what the conceivable or relevant costs and outcomes to be captured are. Consequently, there can be uncertainty regarding which costs should be included, and the way the societal perspective is conceptualised and interpreted can vary [82]. Even studies that state they are using it can omit potentially relevant costs and outcomes, and the societal perspective is often less comprehensive than it could be [8]. This is notable as the choice of its conceptualisation can seriously affect the result of a health economic analysis and the variation in how it is implemented can make comparisons more challenging. More generally, it could be argued that the societal perspective increases the risk of gaming as methods are less standardised, and there are more prominent data gaps [7].

A further consideration surrounding the use of the societal perspective and the variation in its implementation relates to the ongoing debate regarding the inclusion of indirect non-health benefits within economic evaluations (i.e., averted productivity costs) [52, 53, 55]. From a broad utilitarian moral standpoint, including these benefits in economic evaluations is important to ensure the maximisation of the collective benefit to society from the allocation of healthcare resources. However, including productivity gains could lead to the prioritization of the treatment of one group of patients over another because one group generates greater non-health benefits, thereby failing to give equal moral concern and weight to each person’s health care needs. Consequently, there is also a potential moral argument for ignoring productivity gains, in line with Kant's moral theory and that the equitable distribution of healthcare resources should be based on individual health needs [83]. A further factor is that quantifying all relevant non-health outcomes and productivity gains could potentially be double-counting the effectiveness of interventions [53], and this is an area of debate within the field (outlined previously in Box 1). Due to these factors, even under the societal perspective, the inclusion of productivity costs (as well as types of productivity costs) is variable. It is also important to note that productivity costs are particularly sensitive to the methodology used to calculate them, and the different methods used can generate significantly different results (Box 1) [84]. It is vital to consider this variation regarding the types of productivity costs being considered and their calculation within economic evaluations when making comparisons between studies.

A further issue relates to what “society” should be considered under the societal perspective: does “society” refer to the entire world or the society of an individual country. This issue becomes more prominent when evaluating interventions with a limited supply or that involve cross-border issues [22]. Although this can influence to what degree societal costs will be included, it is not always clear what is the scope of the society of interest within studies.

Even if the societal perspective is being used correctly, it can be unclear how the information produced informs choices across different settings and decision-makers—particularly when decision-makers may have different judgements about what outcomes are relevant to their relative values [85]. This is an important limitation for which progress is being made: for example, Walker et al*.* [74] developed a framework for the economic evaluation of policies with the costs and outcomes falling on different sectors (e.g., health, criminal justice, education) and involving different decision makers.

To summarise, while the societal perspective offers some significant advantages, corresponding issues and challenges should also be acknowledged, particularly in LMIC settings. It is worth noting that the societal perspective will not always be required as, ultimately, economic evaluations must align with and serve the stated goals of the decision-maker. In the United Kingdom, the primary focus of the decision-maker is to enhance health outcomes efficiently within a fixed health budget [28, 85]. Therefore, in this context, adopting a health care provider perspective is typically considered more justifiable than a societal perspective. In contrast, this perspective could be misleading in settings where co-payments by the patients are notable, when the goal is to enhance the health system’s efficiency as a whole. Consequently, the choice of the perspective will depend on the purpose of the analysis, who needs to know/use the results and policymakers'/stakeholders’ viewpoints. It is also important to consider that the adoption of the societal perspective can involve notable additional data needs and the corresponding resource needs for collecting this data. This is not to discourage adoption of the societal perspective but rather to highlight that it is not a universal gold standard and the aforementioned factors/challenges are a consideration regarding its adoption.

## Recommendations for policy and research

The terminology used to describe perspectives is variable within the literature. We have endeavoured to use the most established definitions, but it is possible that studies have interpreted and used them differently as well as potentially referred to terminology not included here. We recommend that the global health economic field set more standard definitions of the different perspectives and boundaries between these terms. This is to prevent confusion and misunderstanding not only among researchers but also policymakers and the public as a whole.

A related issue is that broader perspectives consider a wider range of costs, and therefore are likely to capture greater potential cost savings resulting from health interventions. If the healthcare budget is fixed, then this would imply that the cost-effectiveness threshold should be lower for a broader perspective, i.e. that different perspectives should be accompanied by different thresholds. However, the implications go beyond simply lowering the threshold. Some of these cost savings may extend beyond the designated budget holder (e.g. the health care provider), and the presence of budget constraints and trade-offs with other sectors need to be considered. For example, switching from the health care provider perspective to the societal perspective would mean that the provider (e.g. Department of Health) is effectively subsidising other sectors and without increasing the budget, the change could effectively decrease the amount of health being generated. Hence, we recommend that future studies further explore how to more accurately account for the interaction between the chosen perspective and appropriate cost-effectiveness threshold, considering the corresponding impact of budget constraints and trade-offs with other sectors [85]. In addition, not accounting for the impact of the use of different perspectives could potentially lead to biases in decision making, with interventions that have been evaluated with the societal perspective (including more cost savings) more likely to be favoured compared to those that have been evaluated with a narrower perspective.

A factor that needs to be further investigated on the implications of the chosen perspectives is the presence and impact of budget constraints as well as the desired time scale of investment returns.

A key issue to consider when evaluating and interpreting health economic studies is the potential inclusion of productivity costs when using the societal perspective. Estimates of productivity costs are highly sensitive to the method used [56], and it is important to be aware of the potential variation in methodology when comparing studies. In addition, the types of productivity costs included can vary—even when using the societal perspective. We recommend that this is an area that should have more comprehensive and consistent reporting in future studies. Having more standardised productivity cost estimates (potentially within country specific economic evaluation reference cases/guidelines) could be helpful to ensure increased consistency between studies for a particular country setting.

In this paper, we focused on the implications of the perspectives used in economic evaluations such as cost-effectiveness and cost-utility analysis. However, it is also important for future work to consider how the perspective interacts with other health economics methods and frameworks used within public health [86–89].

## Conclusion

When conducting an economic evaluation of a particular intervention, or health technology, the concept of perspective is paramount. There are different types of perspectives which are used in economic evaluations with corresponding differences in the types of costs that are considered, as well as what outcomes are included (for example it can affect if/what “cost savings” are included). The choice of perspective can have a significant impact on the results of economic evaluations. Its relative impact on the results will depend on the context of the study and the intervention being investigated.

When choosing the perspective, it is important to consider the role of patient out-of-pocket payments. Crucially, the payer and health care provider(s) perspectives will not account for any costs paid by patients (including other out-of-pocket payments). They therefore may not be suitable for interventions that require co-payments by patients, as they could underestimate the cost of interventions and potentially lead to inefficient policy recommendations. This is particularly an important consideration in LMIC settings where out-of-pocket payments can be a significant source of health care expenditure [19].

Concerningly, the terminology used to describe the different perspectives is variable within the literature. We have endeavoured to highlight what we consider to be the most established definitions. We recommend that the global health economic field set more standard definitions of the different perspectives and boundaries between these terms.

Finally, it is important to note that despite the advantages of the societal perspective, its adoption does involve additional data needs and there is notable variation in how it is implemented, particularly surrounding what types of productivity costs are considered. Ultimately, there is no universal gold standard regarding what perspective should be used as it depends on the context (including policymakers'/stakeholders’ viewpoints and data/resource availability) as well as the question that the evaluation aims to provide an answer to [7].

## Data Availability

The authors confirm that the data supporting the findings of this study are available within the article.
